# Active Touch Intervention Using a Rough Texture Enhances Corticospinal Excitability

**DOI:** 10.1002/brb3.70837

**Published:** 2025-09-09

**Authors:** Kako Tanabe, Sho Kojima, Kei Saito, Hideaki Onishi

**Affiliations:** ^1^ Graduate School Niigata University of Health and Welfare Niigata Japan; ^2^ Department of Physical Therapy Niigata University of Health and Welfare Niigata Japan; ^3^ Institute for Human Movement and Medical Sciences Niigata University of Health and Welfare Niigata Japan

**Keywords:** active touch intervention, motor‐evoked potential, texture, transcranial magnetic stimulation

## Abstract

**Introduction:**

We aimed to clarify the effects of an active touch intervention using different textures on corticospinal excitability.

**Methods:**

A total of 30 healthy individuals participated in the active touch intervention. Two tactile stimuli were used for intervention: smooth (silk) and rough (hessian) stimuli. Motor‐evoked potentials were measured using transcranial magnetic stimulation to evaluate corticospinal excitability.

**Results:**

The rough‐textured stimulus increased corticospinal excitability 15 min after the intervention. In contrast, the active touch intervention that used a smooth texture did not alter corticospinal excitability.

**Conclusion:**

The occurrence of changes in corticospinal excitability after an active touch intervention may depend on the texture used for the intervention.

## Graphical abstract

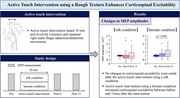

## 1 Introduction

Passive and active touch are input methods that are used to impart tactile stimuli (Gibson 1962; Lederman 1981). Passive touch involves the application of tactile stimuli to the skin without voluntary movement (Ackerley et al. 2012; Pruett et al. 2000), whereas active touch involves the application of tactile stimuli to the skin using voluntary movement (Ackerley et al. 2012; Olczak et al. 2018). Passive touch to the dorsal hand reportedly induces activity in brain regions, including motor and sensory areas, such as the primary motor cortex (M1) and the primary (S1) and secondary (S2) somatosensory cortices (Inui et al. 2004). Moreover, the application of somatosensory stimuli to the skin modulates corticospinal excitability within the cerebral cortex (Kojima et al. 2018; Oliver and Tremblay 2009). Compared with the passive touch intervention, wherein tactile stimulation alone is applied, the active touch intervention, which involves tactile stimulation and fingertip movements, activates a broader range of brain regions in the M1 and S1 (Hinkley et al. 2007); therefore, the active touch intervention has the potential to increase M1 activity and corticospinal excitability considerably.

Active touch involves determining the texture of a stimulus (Lederman and Taylor 1972; Weber et al. 2013). Furthermore, the skin vibrations caused by texture inputs are important for perceiving textures (Hollins and Risner 2000). Textures can be grossly classified as smooth or rough (Weber et al. 2013). Smooth textures (silk or cotton) comprise fine particles (i.e.,  ≤ 200 µm) and are perceived based on the skin vibrations produced when an object is rubbed using the fingertips (Weber et al. 2013; Moungou et al. 2016; Scheibert et al. 2009). Conversely, rough textures (e.g., hessian, grids, and plastic pins) typically comprise particles > 200 µm and provide spatial information about the surface of the stimulus (i.e., textural irregularities, grooves, and shapes) (Lederman and Taylor 1972; Weber et al. 2013; Bauer et al. 2006; Hollins et al. 2002; Tang et al. 2020). A previous study reported differences in brain rhythmic activity after exposure to smooth or rough textures during an active touch intervention. Compared with smooth textures, rough textures elicited a stronger alpha‐band event‐related desynchronization (ERD) in sensorimotor regions (Henderson et al. 2022). The frequency of the alpha‐band rhythm is 8–12 Hz (Foxe and Snyder 2011; Jensen and Mazaheri 2010), and ERD reportedly occurs when processing sensory information or performing exercise (Pfurtscheller 1992). Furthermore, when ERD increases, the amplitude of motor‐evoked potentials (MEPs)—which are indicators of corticospinal excitability—also increases (Takemi et al. 2013a; Takemi et al. 2013b). Therefore, the use of different textures for active touch intervention may alter cortical activity and corticospinal excitability in a texture‐dependent manner. Here, we considered that rough texture may increase corticospinal excitability.

To date, changes in brain rhythms during active touch have been identified in studies using the electroencephalogram. However, whether changes in cortical excitability occur before and after the use of active touch intervention has not been examined. Therefore, this study reports new neurophysiological changes between before and after a new active touch intervention. We aimed to determine the effects of an active touch intervention on corticospinal excitability using two different textures (smooth and rough). Based on previous studies (Henderson et al. 2022), we hypothesized that the cortical activity changes in response to textural differences and that smooth and rough stimuli increase corticospinal excitability. Moreover, we predicted that an active touch intervention using a rough texture would result in greater increases in corticospinal excitability compared with a smooth texture.

## 2 Materials and Methods

### 2.1 Participants

A total of 30 healthy right‐handed individuals (aged 21–24 years; mean ± standard deviation, 22.0 ± 0.9 years; 17 males and 13 females) were recruited into this study. Handedness was assessed using the Edinburgh Handedness Test (mean ± standard deviation, 94.3 ± 10.3). None of the participants had neurological or psychiatric illnesses or were using medications that affected the central nervous system. This study was approved by the Ethics Committee of the Niigata University of Health and Welfare and was conducted in accordance with the principles of the Declaration of Helsinki. Written informed consent was obtained from all participants.

### 2.2 Study Design

**FIGURE 1:**
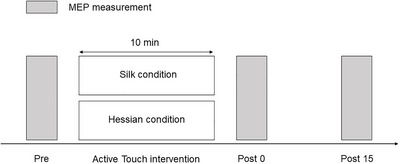
Experimental protocol. Motor‐evoked potentials measurements were performed before, immediately after, and 15 min after the active touch intervention.

The participants were seated comfortably in a chair with a backrest, their palms facing down. During the MEP measurements, the participants were instructed to focus on the target. The MEP measurements were performed before (Pre), immediately after (Post 0), and 15 min after (Post 15) the active touch intervention (Figure 1). The interventions were presented in a randomized order, with at least a 1‐week interval between each condition.

### 2.3 Measurement of Corticospinal Excitability and Electromyography Signals during Active Touch Interventions

Electromyography (EMG) signals were recorded from the right first dorsal interosseous (FDI) muscle using a silver/silver chloride electrode (Zipp 1982). EMG signals were amplified 100‐fold using an amplifier (FA‐DL‐140, 4 Assist, Tokyo, Japan) and recorded using an A/D converter (PowerLab 16/35, AD Instruments, Colorado, USA) at a sampling frequency of 4 kHz. Transcranial magnetic stimulation (TMS) with monophasic pulses was employed to induce MEP using a figure‐8 coil connected to a Magstim 200 square (Magstim Co, Dyfed, UK). The coil was fixed at a 45° transverse tilt to the mid‐sagittal plane. The stimulation site was the hottest MEP evoked area (hotspot) around 6 cm to the left lateral and 2 cm anteriorly from the Cz, according to the international 10–20 system. The optimal coil position was marked on the cap worn by the participant. Moreover, coil position and orientation were monitored throughout the experiment using a Visor2 TMS Neuronavigation System (Emagine Medical Imaging Solutions GmbH, Berlin, Germany). The TMS intensity was set to induce an MEP of approximately 1 mV in the right FDI at rest. The stimuli were presented 20 times, with a stimulation interval of 5–6 s. EMG and MEP amplitudes were recorded and analyzed during the experiment using LabChart 8 (AD Instruments).

### 2.4 Active Touch Intervention

**FIGURE 2:**
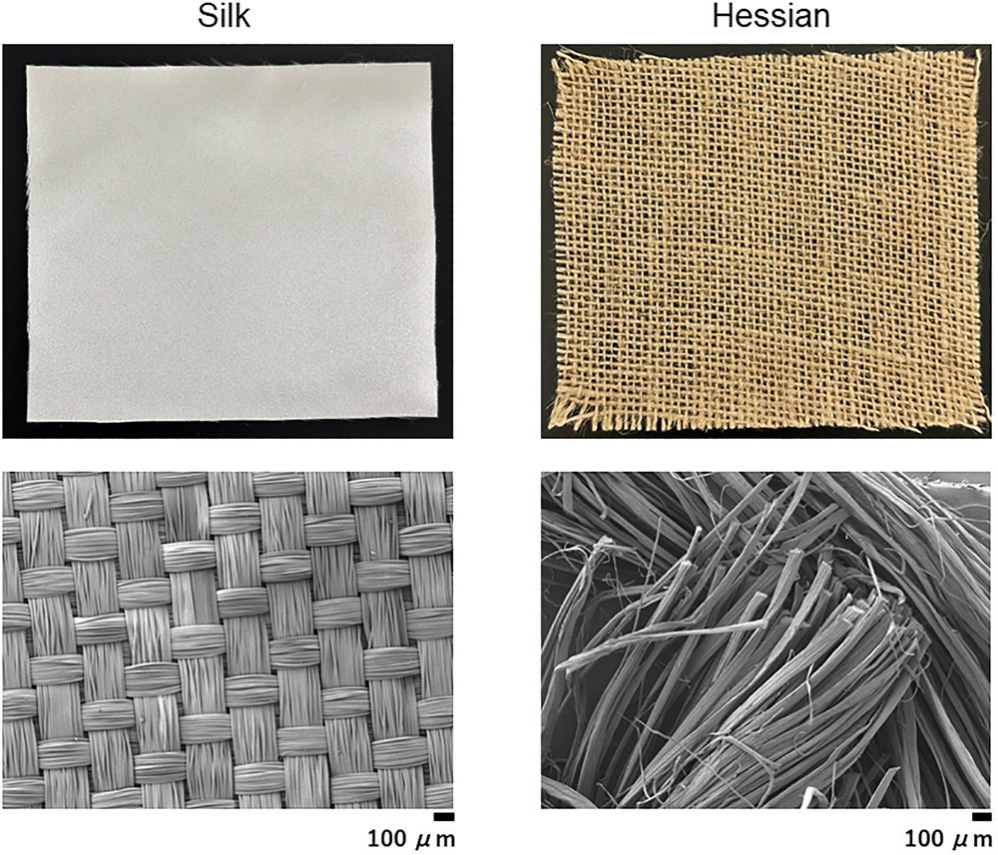
Magnification (50×) of the smooth and rough texture stimuli. Silk (smooth) and Hessian (rough) conditions were used as the tactile stimuli. Each texture (25 mm long and 40 mm wide) was attached to a plastic board with double‐sided tape.

Based on a previous study, smooth (silk) and rough (hessian) tactile stimuli were used in all active touch interventions (Figure 2) (Henderson et al. 2022). The tactile stimuli comprised 25 × 40 mm rectangles that were attached to a plastic board using double‐sided tape. All active touch interventions lasted 10 min and involved voluntary and repeated right index finger adduction/abduction movements of the right index finger (0°–10°). The exercise frequency was set to 0.5 Hz, as indicated by metronome pacing. The participants performed 300 repetitions during each active touch intervention (Watanabe et al. 2020). Blocks were placed at both ends of the presented texture rectangle. Each participant was instructed to slightly move their right index finger as quickly as possible over the textured rectangle until it hit the block without pressing the object. Moreover, they were asked to maintain a forward gaze and lightly rub the textured sample. An accelerometer (FA‐DL‐111A, 4 Assist, Tokyo, Japan) was affixed to the right proximal interphalangeal joint to measure acceleration and muscle activity during the intervention.

### 2.5 Subjective Ratings

Subjective ratings based on a Visual Analog Scale were obtained in writing immediately after the active touch intervention. The participants were asked to mark a point on a 100‐mm line drawn on paper. The four evaluation items were “strongly felt or not felt at all,” “unpleasant or pleasant,” “rough or smooth,” and “hard or soft” (Henderson et al. 2022).

### 2.6 Statistical Analysis

The MEP amplitude was calculated as the peak‐to‐peak amplitude of the additive mean of 20 trials of each type (Pre, Post 0, Post 15). No trials were excluded from the MEP measurements performed in this study. EMG activity during the active touch intervention was band‐pass filtered (20–500 Hz), full‐wave rectified, smoothed (501 points), and averaged using the right index finger adduction/abduction movement as one interval. Muscle activity was monitored constantly, and the absence of muscle activity during rest periods and MEP measurements was noted. Subsequently, the EMG activity was averaged during the intervention for each movement. For the maximum adduction/abduction acceleration of the right index finger during the active touch intervention, each motion was considered as one interval, and the maximum (abduction movement) and minimum (adduction movement) acceleration values in the *x*‐axis direction for each movement interval were calculated. Finally, the mean acceleration value for each section interval was calculated.

Statistical analyses were conducted using the SPSS Statistics 27 software (IBM, Armonk, New York, USA). The Shapiro–Wilk test was used to test the normality of the data, whereas Wilcoxon's signed rank test was employed to compare the TMS intensity values. Furthermore, this study had a within‐subjects design. Time was factored, and Friedman tests were used to compare MEP amplitude values before, immediately after, and 20 min after the intervention in each condition. The MEP amplitude values for each condition were compared using the Shapiro–Wilk test as a test of normality, and because the data did not follow normality in the hessian and silk conditions, the Friedman test, a nonparametric test, was performed. All post hoc tests were performed using Wilcoxon's signed rank test, then adjusted using the Bonferroni method. Subjective ratings for each condition were compared using Wilcoxon's signed rank test. Comparisons of muscle activity and maximum abduction/adduction acceleration during the active touch intervention for each condition were performed using Wilcoxon's signed rank test. The significance level was set at 5%.

## 3 Results

### 3.1 Comparison of MEP Amplitudes

The mean TMS intensities were 63.4 ± 12.0% (mean ± standard deviation) for the smooth texture conditions and 62.9 ± 11.3% for the rough texture conditions. Wilcoxon's signed rank test revealed the absence of significant between‐group differences in stimulus intensity (*p* = 0.914).

**FIGURE 3:**
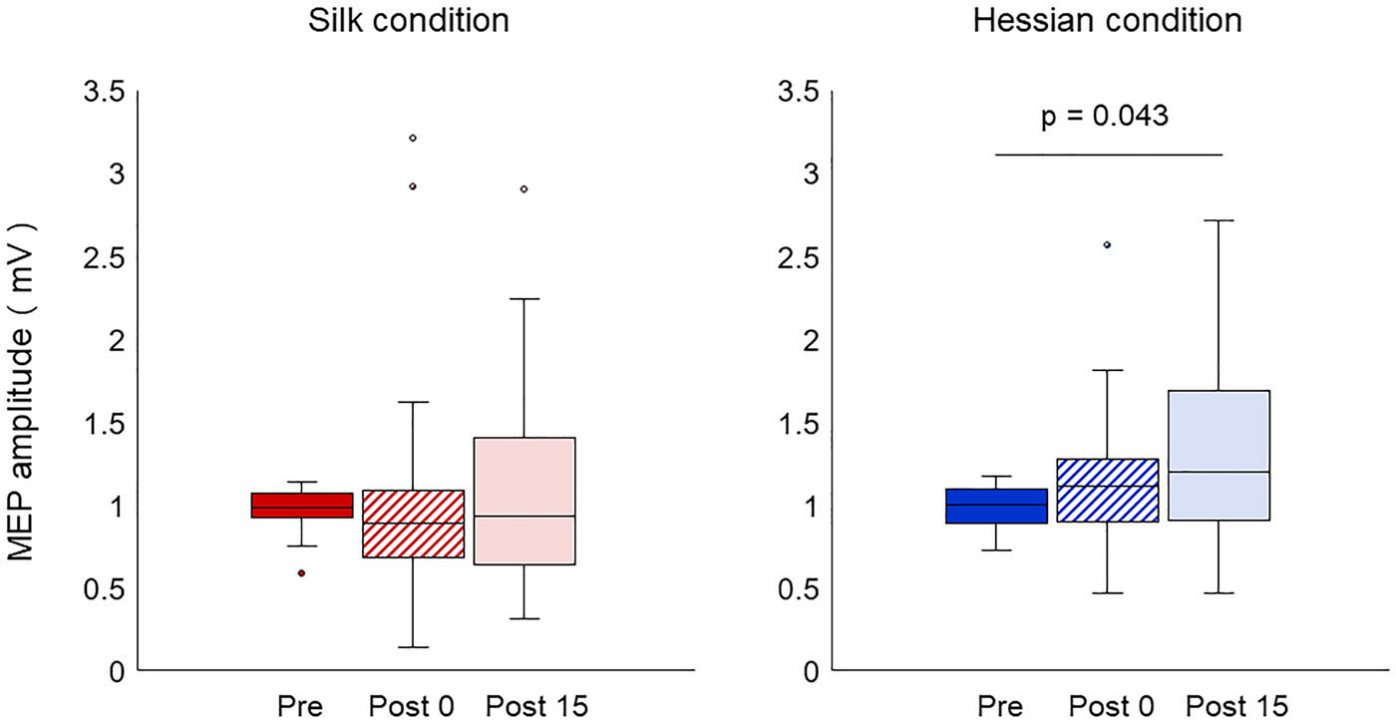
Motor‐evoked potentials (MEP) amplitudes under both conditions before and after the active touch intervention. No statistically significant differences in corticospinal excitability were noted between before and after the active touch intervention using a smooth stimulus (*p* = 0.587). When a rough stimulus was used, a significant increase in MEP amplitude values was observed at Post 15 compared with Pre (*p* = 0.043).

The silk condition had 2 degrees of freedom, a sample size of 30, and a statistical value of 1.067. The mean MEP amplitude values for the smooth texture conditions were 0.98 ± 0.02 mV (Pre), 1.01 ± 0.12 mV (Post 0), and 1.07 ± 0.11 mV (Post 15). The Friedman test indicated the absence of statistically significant differences in corticospinal excitability between before and after the active touch intervention using a smooth stimulus (*p* = 0.587). In contrast, in the active touch intervention using a rough stimulus, the hessian condition had 2 degrees of freedom, a sample size of 30, and a statistical value of 6.067. The mean MEP amplitude values were 0.99 ± 0.02 mV (Pre), 1.14 ± 0.08 mV (Post 0), and 1.33 ± 0.11 mV (Post 15). Friedman's test revealed a statistically significant difference in corticospinal excitability between before and after the active touch intervention using a rough stimulus (*p* = 0.048). Compared with Pre, a significant increase in MEP amplitude values was noted at Post 15 (*p* = 0.043, Wilcoxon's signed rank test with Bonferroni corrections). There was no significant difference in MEP amplitude between Pre and Post 0 or between Post 0 and Post 15 (Pre–Post 0, *p* = 0.905; Post 0–Post 15, *p* = 0.467) (Figure 3). Furthermore, a comparison of the MEP amplitude values measured in the two Pre conditions was performed using the Shapiro–Wilk test, which did not indicate normality (hessian condition: *p* = 0.055; silk condition: *p* = 0.010); therefore, a Wilcoxon signed rank test was performed, which showed no significant differences between the conditions (*p* = 0.766).

### 3.2 Comparison of Subjective Evaluations

**FIGURE 4:**
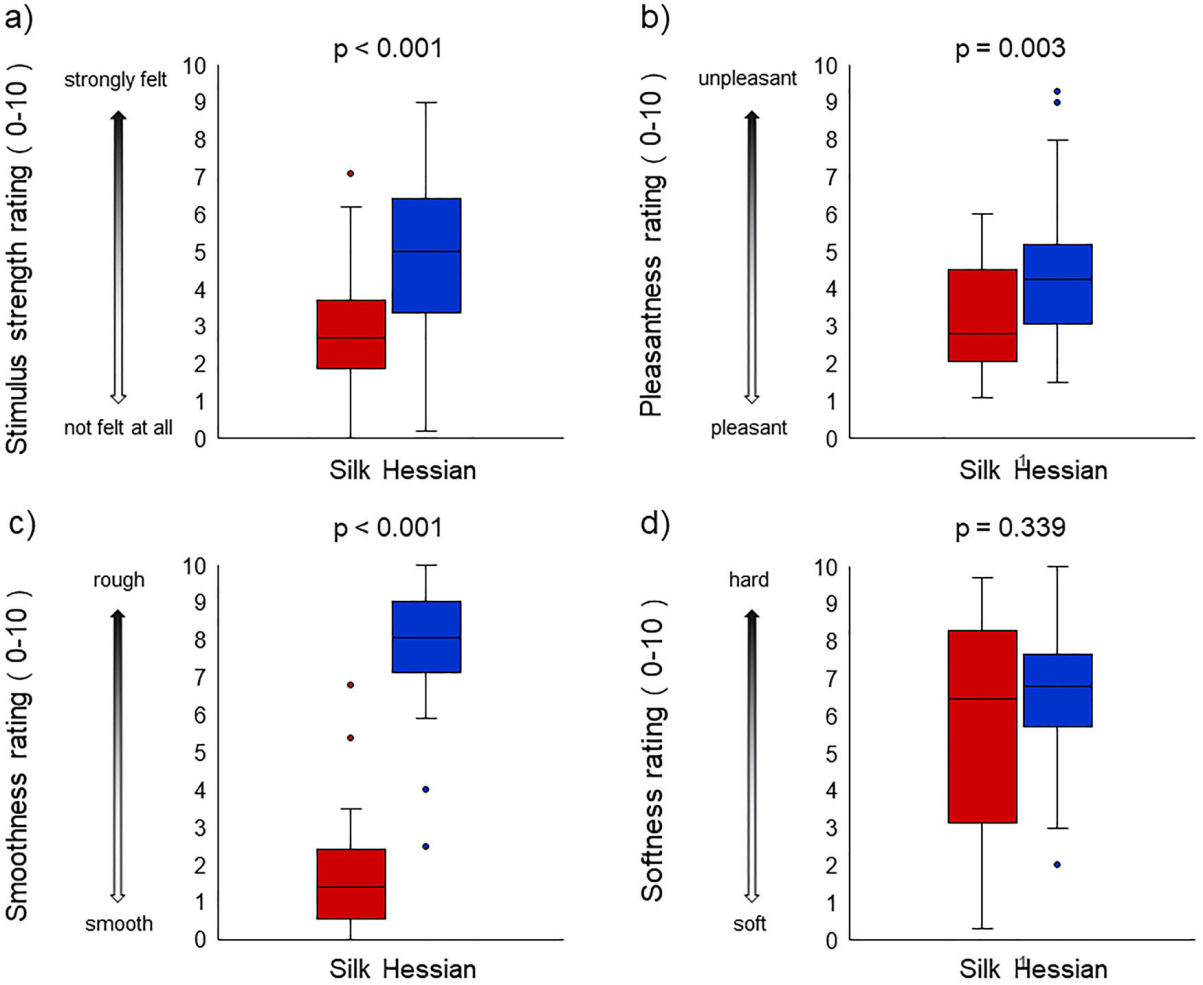
Subjective ratings of the smooth and rough textures. When a rough stimulus was used, the participants perceived the stimulus as being stronger (*p* < 0.001), more unpleasant (*p* = 0.003), and rougher (*p* < 0.001) compared with the smooth condition. We observed no significant difference in perceived softness between the smooth and rough conditions (*p* = 0.339).

Wilcoxon's signed rank test revealed that the perceived intensity of the texture stimuli was significantly stronger for the rough stimulus compared with the smooth stimulus (*p* < 0.001). Moreover, the stimulus texture was significantly unpleasant (*p* = 0.003) and rough (*p* < 0.001) when the rough stimulus was used. However, there was no significant difference in the perceived “hardness” or “softness” between the two conditions (*p* = 0.339) (Figure 4).

### 3.3 Comparison of Maximum Adduction/Abduction Acceleration and Muscle Activity during the Active Touch Intervention for Each Condition

Wilcoxon's signed rank tests revealed the absence of significant differences in the maximum abduction (*p* = 0.339) and adduction (*p* = 0.572) accelerations or muscle activity (*p* = 0.339) between the two conditions (Table 1).

**TABLE 1:** Maximum abduction/adduction acceleration and muscle activity during active touch intervention using smooth and rough stimuli. Maximum abduction/adduction acceleration and muscle activity during active touch intervention were compared between smooth and rough conditions. There was no significant difference in maximum abduction (*p* = 0.339) or adduction (*p* = 0.572) acceleration and muscle activity (*p* = 0.339) during active touch intervention between the two conditions.

	Smooth stimulus	Rough stimulus	*p*
Maximum abduction acceleration (m/s^2^)	0.34 ± 0.25	0.39 ± 0.23	0.339
Maximum adduction acceleration (m/s^2^)	−0.24 ± 0.27	−0.27 ± 0.21	0.572
EMG activity (mV)	0.03 ± 0.02	0.02 ± 0.02	0.339

*Note*: Values are expressed as mean ± standard division; abduction, +; adduction, −.

## 4 Discussion

This study showed that an active touch intervention using a rough stimulus increased corticospinal excitability considerably compared with the preintervention condition. However, when a smooth stimulus was used, there was no significant change in corticospinal excitability before and after the active touch intervention. Moreover, the rough stimulus was perceived as being stronger, coarser, and more unpleasant than the smooth stimulus. These results suggest that active touch interventions affect corticospinal excitability differently according to the texture of the stimulus.

The active touch intervention using a rough stimulus increased corticospinal excitability 15 min after the intervention. This result may be related to the rhythmic activity in the alpha band that occurs after the active touch intervention. Alpha‐band ERD occurs in the sensorimotor area contralateral to the stimulus input during active touch when a rough stimulus is used (Henderson et al. 2022). In addition, alpha‐band ERD has been associated with a reduced power of brain rhythms and increased cortical excitability (Neuper and Pfurtscheller 2001; Pfurtscheller 2001; Pfurtscheller and Aranibar 1977). Furthermore, the lower alpha power recorded in the M1 during the vibrotactile discrimination task indicates higher neuronal firing rates (Haegens et al. 2011), and a decreased alpha power indicates increased cortical excitability in the brain regions associated with the task (Haegens et al. 2011; Yamagishi et al. 2003). Therefore, it is possible that corticospinal excitability—including M1 excitability—was significantly increased during the active touch intervention when a rough stimulus was used.

The alpha‐band ERD increases considerably when attention is directed spontaneously to tactile stimuli (Silas et al. 2019). Attempting to identify an object by moving a fingertip across the stimulus considerably increases corticospinal excitability compared with an attempt to identify an object by moving the finger without paying attention to the object (Master and Tremblay 2009). Based on a previous study (Silas et al. 2019; Master and Tremblay 2009), we considered that the rough stimulus may have increased the participant's attention during the active touch intervention. The increases in corticospinal excitability observed after exposure to a rough stimulus may have resulted from this enhancement in attention to the tactile stimuli.

The active touch intervention using a smooth stimulus yielded no significant changes in corticospinal excitability between before and after the intervention. Smooth‐textured silk comprises fine fibers (Hollins and Risner 2000) and a texture (Weber et al. 2013) with small spatial texture irregularities (Hollins et al. 2002). Previous studies have reported a temporary decrease in M1 excitability after repeatedly performing a voluntary exercise (Bonato et al. 2002). This phenomenon is known as postexercise depression (PED) and occurs after light‐load repetitive exercise (Miyaguchi 2017). Based on these previous findings, we concluded that an active touch intervention using a smooth stimulus may not sufficiently counteract the PED caused by repetitive voluntary movements to increase corticospinal excitability significantly.

In the present study, the MEP amplitude values increased significantly 15 min after the intervention, rather than immediately after it, in the hessian condition. Moreover, similar to previous studies reporting that increases in M1 excitability are enhanced after the occurrence of increases in S1 excitability (Brinkman et al. 1985), changes in corticospinal excitability occurred after a delay. Therefore, in this study, it is possible that the active touch intervention in the hessian condition increased S1 activity, followed by an increase in M1 excitability, resulting in a delayed increase in MEP amplitude values at 15 min after, rather than immediately after, the intervention.

The rough condition was perceived as more irritating, rougher, and unpleasant compared with the smooth condition. Rough textures convey information about a texture's unevenness (Hollins et al. 2002). Furthermore, rough textures are perceived as unpleasant when touched or rubbed (Klöcker et al. 2013). Therefore, it is possible that the rough burlap conveyed a stronger, rougher, and more unpleasant sensation because of the thicker fibers and uneven composition of the stimulus. However, there was no significant difference in the perceived hardness between the smooth and rough stimuli. The perceived hardness of the tactile stimuli is based on the pressure information (Dhong et al. 2019). This study used a task in which the participants rubbed a texture using their fingertips and were instructed to move their right index finger without pressing the stimulus object. This could explain the lack of between‐group differences in perceived hardness.

One of the limitations of this study was that it remains unclear whether the active touch intervention using a rough stimulus reduced the alpha‐band power. Such changes in alpha‐band brain rhythms can be measured using electroencephalography before and after the administration of the active touch intervention. Moreover, it remains unclear whether the increases in corticospinal excitability observed here were caused by changes in voluntary movement combined with tactile stimulation or by exposure to a single texture. Future studies should establish conditions involving passive exposure to different textures during active movement.

## 5 Conclusion

Active touch intervention using a rough‐textured stimulus increased corticospinal excitability between before and after the intervention, whereas no changes in corticospinal excitability were noted after the active touch intervention using a smooth stimulus.

## Author Contributions

**Kako Tanabe**: conceptualization, writing – original draft, investigation, methodology, formal analysis. **Sho Kojima**: conceptualization, methodology, funding acquisition, writing – original draft. **Kei Saito**: writing – original draft. **Hideaki Onishi**: funding acquisition, writing – original draft, supervision.

## Ethics Statement

This study was approved by the Ethics Committee of the Niigata University of Health and Welfare and conducted in accordance with the principles of the Declaration of Helsinki.

## Consent

Written informed consent was obtained from each participant for the experiment.

## Conflicts of Interest

The authors declare no conflicts of interest.

## Peer Review

The peer review history for this article is available at https://publons.com/publon/10.1002/brb3.70837.

## Data Availability

Data will be provided upon request.
